# Salivary Alpha-Amylase Activity Levels in Catatonic Schizophrenia Decrease after Electroconvulsive Therapy

**DOI:** 10.1155/2018/2623585

**Published:** 2018-05-10

**Authors:** Misako Kanayama, Tsuyoshi Miyaoka, Tomoko Araki, Maiko Hayashida, Sadayuki Hashioka, Jun Horiguchi

**Affiliations:** Department of Psychiatry, Shimane University of Medicine, 89-1 Enyacho, Izumo 6938501, Japan

## Abstract

**Background:**

Dysfunction of the autonomic nervous system (ANS) in schizophrenia has been detected by electrophysiological methods, but the underlying mechanisms remain unknown. Several studies have suggested that measuring salivary alpha-amylase activity levels is useful for evaluating the ANS activity and that sAA levels increase in schizophrenia and correlate with Brief Psychiatric Rating Scale (BPRS) scores. However, no study has examined the relationship between sAA activity levels and symptoms of schizophrenia with catatonic state.

**Methods:**

We present the case of a 59-year-old female with persistent catatonic schizophrenia treated by electroconvulsive therapy. We evaluated the ANS activity by measuring sAA activity levels before and after ECT, and we evaluated her symptoms using the BPRS and Bush–Francis Catatonia Rating Scale (BFCRS).

**Results:**

ECT was highly effective and BPRS and BFCRS scores substantially decreased. sAA activity levels decreased from 125 kU/l to 33 kU/l.

**Conclusions:**

sAA activity levels could be a potential biomarker of schizophrenia with catatonic state.

## 1. Introduction

The pathology of schizophrenia remains unknown, and a relevant biomarker remains to be identified. Various studies have been conducted to address this, including studies on the autonomic nervous system (ANS) [1–7]. These studies have indicated a dysfunction of the ANS in patients with schizophrenia [1, 2, 5–8].

Measurement of salivary alpha-amylase [9] activity levels is reportedly useful for evaluating activity of the ANS [10–13], and a simple, noninvasive appliance has been recently developed to measure sAA activity levels [14, 15]. sAA activity levels of patients with schizophrenia have been reported to be higher than those of controls, and these levels have been associated with the severity of symptoms in patients with schizophrenia [16, 17].

However, only one study has examined the change in diachronic sAA activity levels before and after treatment [18]: it was a case report of a patient treated with electroconvulsive therapy [19] for schizophrenia; however, only one psychiatric symptoms rating scale, the Brief Psychiatric Rating Scale (BPRS), was used. Using ECT, the symptoms of catatonia are easier to heal than the other symptoms of schizophrenia; thus, we examined Bush–Francis Catatonia Rating Scale (BFCRS). To the best of our knowledge, no previous report has checked the relationship between sAA activity levels and BFCRS.

Here, we discuss the case of a patient with catatonic schizophrenia treated by ECT. Because persistent catatonia was an important element, we assessed her symptoms using the BFCRS, a gold standard scale for catatonia [20–23], and examined the relationship between sAA activity levels and BFCRS scores for catatonia before and after treatment.

## 2. Case Presentation

A 59-year-old woman was admitted to our psychiatric department for ECT. She had been diagnosed with schizophrenia at the age of 17 years and had taken antipsychotic drugs for 42 years. She had no history of alcohol consumption, drug use, or epileptic seizures. One day, at the age of 55 years, she lay on a road and was unable to move or speak due to a catatonic state. She was immediately admitted to a hospital. However, medicines proved ineffective, despite attempting several types of second-generation antipsychotic drugs and benzodiazepines. Her white blood-cell counts were too low for clozapine use. Thus ECT was recommended, and she was transferred to our hospital, the only hospital in the area to offer ECT.

She exhibited severe catatonia as well as many other psychotic symptoms, such as hallucination, delusion, and negative symptoms. She was taking risperidone 9 mg daily without side effects for nearly 10 years. Besides she had been taking magnesium oxide 1.2 g and sennoside 36 mg daily for constipation for many years. The minor tranquilizer was stopped for ECT and was no longer necessary after ECT. We performed ECT, applied three times a week for a total of 14 sessions. Thiopental and ketamine were used to induce anesthesia.

We evaluated her symptoms by BPRS and BFCRS (23 items) at 14 and 7 days before ECT and after the course of ECT was complete. On the same days, we measured her sAA activity levels with a colorimetric salivary biosensor (Nipro Co., Japan). In order to eliminate the effects of hospitalization, we measured sAA activity levels again not only immediately after hospitalization but also just before ECT. Thus, we measured her sAA activity levels twice before ECT. The saliva samples were collected in the morning following a 10-min rest on a chair or bed, at least 2 h after the last meal and tooth-brushing.

**Figure 1:**
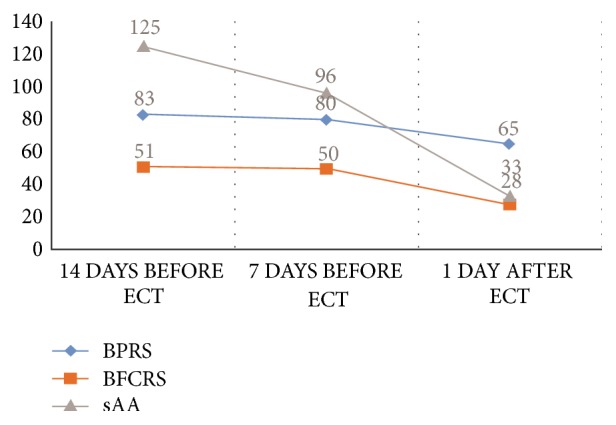
The relationship between the patient's salivary alpha-amylase activity levels (kU/l) and scores for the Brief Psychiatric Rating Scale (BPRS) and Bush–Francis Catatonia Rating Scale (BFCRS).

ECT was extremely successful, and her symptoms considerably improved after the 14 sessions, with remarkable reductions in BPRS and BFCRS scores. Her BPRS and BFCRS scores decreased from 83 to 65 and from 51 to 28, respectively. Her sAA activity levels also substantially decreased from 125 kU/l to 33 kU/l.  Figure 1 shows the changes in the BPRS, BFCRS, and sAA activity levels before and after ECT.

## 3. Discussion

Here, we investigated the ANS of a patient with persistent catatonic schizophrenia before and after ECT by measuring sAA activity levels. This decreased in a similar manner to the improvement in the BPRS and BFCRS scores before and after ECT. To the best of our knowledge, this is the first report to examine the relationship between sAA activity levels and BFCRS.

Many studies have investigated the ANS, using various electrophysiological methods such as electrodermal measures, heart rate analysis, and measuring blood or salivary cortisol levels [3, 4, 8, 24, 25]. Cortisol is similar to sAA activity levels in reflection to stress. Measuring blood cortisol involves needle prick. Therefore, great stress of pricking needles at the time of blood collection was mixed as a bias to stress of psychosis itself. Salivary cortisol is too difficult to collect from patients with severe psychiatric symptoms. In this study, we used sAA activity levels to evaluate the ANS. Every measurement method has advantages and disadvantages, but sAA, which can be measured easily and quickly, is very useful when the mental symptoms are badly cooperative like this time.

The sAA activity levels are considered to be a noninvasive biomarker for assessing mental stress [11–13, 26, 27], and it has been suggested that it reflects the activity of the sympathetic–adrenal–medullary system [12, 26, 28–31]. Blood pressure, heart rate, plasma catecholamine level, and activities of the sympathetic nervous system in heart rate variability have been shown to correlate with sAA activity levels [32, 33]. However, the use of sAA activity levels has its weaknesses. There have been disagreements over whether high sAA activity reflects the loss of vagal activity or high activity of the sympathetic nervous system [28, 34]. In either case, it is believed to represent a function of sympathetic nerve activity. Beta-blockers, which block the sympathetic nervous system, have been shown to prevent fluctuations in sAA activity levels [31], again suggesting that sAA activity represents a function of sympathetic nerve activity. There has also been some controversy over how to collect saliva [34–37]. For the monitor used in this study, saliva was collected using a paper filter, with the sAA activity level on the paper measured immediately. Overall, this has been considered a reliable method [14, 15, 36].

Various previous studies have suggested a dysfunction of the ANS of patients with schizophrenia [4–7, 25]. However, the underlying mechanisms remain unclear. Bar et al. measured heart rate variability and reported that patients with acute schizophrenia showed dysfunction of the ANS activity, particularly parasympathetic activity [2, 8]. Fujibayashi et al. also indicated that there may be depressed activity of the ANS in patients with schizophrenia [38]. Recently, meta-analysis of autonomic nervous function by heart rate variability (HRV) in patients with mental illness was performed [1]. In this study, 1692 patients with psychiatric disorders, including schizophrenia, and 1639 controls were analyzed. Of the patient group, 812 people were not medicated, and 880 were medicated. As a result, it was confirmed that autonomic nervous function was declining dominantly in psychiatric disorders patients under nonmedication compared with healthy group. They concluded that the decline of the autonomic nerve was observed in schizophrenia, regardless of medicine. Our report, although the method used was different, had the same result as these results.

In addition, our colleague observed high sAA levels in such patients [17] and showed that sAA activity levels were proportional to the severity of psychosis, as measured by the BPRS [16, 39]. Our case supported this finding. In other words, the reason why sAA activity levels were high before ECT this time could be because schizophrenia symptoms were severe.

Several studies have investigated the effect of medication on the activity of the ANS [40–43]. However, in our case, there was no change in the medication taken by our patient before and after ECT. In addition, she did not take other antipsychotics or minor tranquilizers. The laxative that she was taking was not changed before and after the ECT. To the best of our knowledge, no studies have been performed suggesting that larvae affect autonomic nerves. Therefore, further examination might be required.

This patient experienced a decrease in the BFCRS score following ECT, and at the same time, her sAA activity levels decreased from high to normal. This might suggest that the sAA activity levels were related to the severity of symptoms of catatonia. Furthermore, our patient's catatonia had been persistent, lasting more than 4 years. If a long-standing disorder of the ANS induced an irreversible change in the nervous systems, sAA activity levels would not be expected to change when symptoms were relieved by ECT. Our findings therefore demonstrated that no irreversible change had occurred as a result of the patient's persistent dysfunction of the ANS. The results of our case support the previous review [6]. That is, autonomic abnormality of schizophrenia may be due to stress caused by the symptoms rather than the disease itself.

There is one report about the relationship between sAA activity levels and the psychiatric symptoms of schizophrenia [18]. In that report, sAA activity levels declined with the improvement of mental symptoms, and it is same as our report, which indicates reproducibility. Additionally, in the present case, the patient experienced a decrease in the BFCRS score following ECT, and concurrently her sAA activity levels decreased from high to normal.

We have discussed one curious thing. Between 14 days and 7 days before the ECT, sAA activity levels decreased intensely though BPRS and BFCRS scores decreased slightly. It was intriguing because measurement conditions at 14 days and 7 days were completely unchanged. It might reflect the gradually declined stress of being transferred from other hospitals to our hospital in addition to ANS disorder due to psychic symptoms themselves. We need to investigate by collecting more cases.

This report has some limitations. We examined the ANS only by measuring sAA activity levels, although heart rate variability is also a useful method. It is unclear whether sAA change was caused by improvement of catatonia, improvement of psychosis, or effect of ECT. There were several studies on the influence of ECT on ANS (no study by sAA). According to them, parasympathetic nerves were stimulated by ECT [44–47]. It was also pointed out that strength of stimulation of ANS may be related to the effect of ECT [46]. However, those studies investigated the impact of several minutes after ECT. As far as we examined, we could not find a study that examined the effects of the following day as our case. Those studies showed as limitations that some of anesthetic drugs were said to stimulate sympathetic nerves and others to stimulate parasympathetic nerves [46, 48]. However, since each drug had a half-life of several hours, it could be hard to think that it continued to exert influence on our study, the following day as well. Next limitation is that our report discusses a single case and only one disease. Further research is needed to investigate the pathology and establish biomarkers of schizophrenia with catatonic state.

We reported the association among sAA activity levels, schizophrenia symptoms, and catatonia in only one case. Our findings suggested that sAA activity level might be a potential biomarker for catatonic schizophrenia.
